# Prox1 Is a Marker for AII Amacrine Cells in the Mouse Retina

**DOI:** 10.3389/fnana.2017.00039

**Published:** 2017-05-05

**Authors:** Luis Pérez de Sevilla Müller, Shaghauyegh S. Azar, Janira de los Santos, Nicholas C. Brecha

**Affiliations:** ^1^Departments of Neurobiology, Medicine and Ophthalmology, David Geffen School of Medicine at Los Angeles, University of California, Los AngelesLos Angeles, CA, USA; ^2^Stein Eye Institute, David Geffen School of Medicine at Los Angeles, University of California, Los AngelesLos Angeles, CA, USA; ^3^CURE Digestive Diseases Research Center, David Geffen School of Medicine at Los Angeles, University of California, Los AngelesLos Angeles, CA, USA; ^4^Veterans Administration Greater Los Angeles Health SystemLos Angeles, CA, USA

**Keywords:** Prox1, transcription factor, glycine, AII amacrine cells, mouse retina, vision

## Abstract

The transcription factor Prox1 is expressed in multiple cells in the retina during eye development. This study has focused on neuronal Prox1 expression in the inner nuclear layer (INL) of the adult mouse retina. Prox1 immunostaining was evaluated in vertical retinal sections and whole mount preparations using a specific antibody directed to the C-terminus of Prox1. Strong immunostaining was observed in numerous amacrine cell bodies and in all horizontal cell bodies in the proximal and distal INL, respectively. Some bipolar cells were also weakly immunostained. Prox1-immunoreactive amacrine cells expressed glycine, and they formed 35 ± 3% of all glycinergic amacrine cells. Intracellular Neurobiotin injections into AII amacrine cells showed that all gap junction-coupled AII amacrine cells express Prox1, and no other Prox1-immunostained amacrine cells were in the immediate area surrounding the injected AII amacrine cell. Prox1-immunoreactive amacrine cell bodies were distributed across the retina, with their highest density (3887 ± 160 cells/mm^2^) in the central retina, 0.5 mm from the optic nerve head, and their lowest density (3133 ± 350 cells/mm^2^) in the mid-peripheral retina, 2 mm from the optic nerve head. Prox1-immunoreactive amacrine cell bodies comprised ~9.8% of the total amacrine cell population, and they formed a non-random mosaic with a regularity index (RI) of 3.4, similar to AII amacrine cells in the retinas of other mammals. Together, these findings indicate that AII amacrine cells are the predominant and likely only amacrine cell type strongly expressing Prox1 in the adult mouse retina, and establish Prox1 as a marker of AII amacrine cells.

## Introduction

Amacrine cells are the most diverse group of cells within the mammalian retina with more than 40 types, distinguished by their size, axonal and dendritic architecture and neurotransmitter content (for reviews see Wässle and Boycott, 1991; MacNeil and Masland, 1998; MacNeil et al., 1999; Masland, 2001, 2012). In the inner nuclear layer (INL), amacrine cells form a band that is 2–3 cell bodies wide along the distal margin of the inner plexiform layer (IPL); they comprise ~41% of all cells in the mouse INL (Strettoi and Masland, 1995; Jeon et al., 1998). Additionally, amacrine cells have been shown to make up ~60% of the neurons in the ganglion cell layer (GCL) of the mouse retina (Jeon et al., 1998).

The majority of amacrine cells contain GABA or glycine immunoreactivity (Vaney, 1990; Menger et al., 1998), while the neurotransmitter identity of ~10% of the amacrine cells is unknown (Kay et al., 2011). GABA-immunoreactive amacrine cells are characterized by medium and wide-field processes, and several wide-field types contain a second neuroactive substance, including vasoactive intestinal polypeptide, substance P, acetylcholine, or dopamine (Brecha et al., 1988; Wässle and Chun, 1988; Vaney et al., 1989; Casini and Brecha, 1992; Strettoi and Masland, 1996; Akrouh and Kerschensteiner, 2015; Park et al., 2015). In contrast, the glycine-immunoreactive amacrine cells have narrow-field processes that span multiple IPL laminae, and several types also contain a second immunohistochemical marker, including parvalbumin, calretinin and Disabled 1 (Wässle et al., 1993; Haverkamp and Wässle, 2000, 2004; Rice and Curran, 2000; Lee et al., 2006, 2016). An exception to this general principle is that VGluT3-immunoreactive amacrine cells, which have medium-field processes distributed to multiple IPL laminae, exhibit both glycine and glutamate immunoreactivity (Haverkamp and Wässle, 2004; Johnson et al., 2004; Grimes et al., 2011; Kim et al., 2015).

AII amacrine cells are among the best-characterized amacrine cell types in the mammalian retina (Kolb and Famiglietti, 1974; Famiglietti and Kolb, 1975; Pourcho and Goebel, 1985; Vaney, 1990; MacNeil and Masland, 1998; Menger et al., 1998; Shen and Jiang, 2007). They are bistratified, narrow–field, glycine-containing amacrine cells that connect rod and cone photoreceptor pathways to transfer visual information from rod photoreceptors to ganglion cells (Demb and Singer, 2012). They are easily recognized by the presence of thick lobular appendages in the OFF sublamina of the IPL and descending arboreal processes to the ON sublamina of the IPL (Famiglietti and Kolb, 1975). They receive input from rod bipolar cells, while providing output onto ON cone bipolar cells through gap junctions. They also provide output onto OFF-cone bipolar cells and OFF-ganglion cells through conventional inhibitory glycinergic synapses (Kolb and Famiglietti, 1974; Strettoi et al., 1992; Chun et al., 1993; Grünert and Wässle, 1996; Hartveit and Veruki, 2012).

Multiple experimental approaches have been used to identify AII amacrine cells in the retina, including the uptake of fluorescent dyes (Vaney, 1985; Mills and Massey, 1991; Vaney et al., 1991; Bloomfield and Völgyi, 2004). Another approach identifying AII amacrine cells is using immunohistochemistry with antibodies to parvalbumin in rabbit (Casini et al., 1995) and rat (Wässle et al., 1993), while antibodies to calretinin have been used in cat (Pasteels et al., 1990; Gábriel and Straznicky, 1992; Macneil et al., 2009), macaque (Wässle et al., 1995; Massey and Mills, 1999; Kolb et al., 2002), rabbit (Massey and Mills, 1999) and human (Lee et al., 2004, 2016). Lastly, disabled 1 has been used in mouse (Rice and Curran, 2000; Lee et al., 2004, 2006). AII amacrine cells have also been labeled in transgenic mouse lines (Vuong et al., 2015). In the mouse retina, AII amacrine cells have only been identified immunohistochemically using antibodies to Disabled 1 (Rice and Curran, 2000; Lee et al., 2004, 2006), and Prox1 immunostaining was stated to label AII amacrine cells in the adult mouse retina (Keeley et al., 2014), but this was not further investigated in that study.

The homeobox gene *prox1* encodes for the transcription factor Prox1, which consists of two main domains, the prospero domain and the homeodomain (Oliver et al., 1993; Bürglin, 1994). This transcription factor regulates proliferation of retinal progenitor cells, and is required for horizontal cell development and bipolar cell differentiation (Cook, 2003; Dyer et al., 2003). Prox1 immunoreactivity is present during the embryonic and postnatal periods in the mouse, rat and human retina (Dyer et al., 2003). During the embryonic period, Prox1 immunoreactivity is exhibited in the outer neuroblastic layer; during the postnatal period, it is present in horizontal, bipolar and amacrine cells in the mouse, rat and chick retina (Belecky-Adams et al., 1997; Dyer et al., 2003). Prox1 immunoreactivity is found broadly in the INL of the adult mammalian retina, specifically in horizontal cells, and in some types of bipolar and Müller cells (Dyer et al., 2003; Cid et al., 2010). Amacrine cells have also been shown to express Prox1 immunoreactivity. In the adult mouse retina, Prox1 immunoreactivity was reported in some calbindin and calretinin immunostained amacrine cells (Cid et al., 2010). In rat retina, Prox1 immunoreactivity was found in AII amacrine cells (Dyer et al., 2003).

In the present study, we have evaluated Prox1 immunostaining in the adult mouse retina with a focus on Prox1 expression in amacrine cells. Prox1 immunoreactivity was strongly expressed in AII amacrine cell bodies in all retinal regions, in contrast to a previous report (Cid et al., 2010). The Prox1-immunoreactive/AII amacrine cells comprise ~10% of the amacrine cell population and they form a non-random mosaic, similar to AII amacrine cells in other mammalian species. Consistent with earlier studies (Dyer et al., 2003; Cid et al., 2010), we also found strong Prox1 immunostaining in horizontal cells and weak immunostaining in bipolar cells.

## Materials and Methods

### Animal Preparation

These studies were conducted under protocols approved by the University of California at Los Angeles (UCLA) Animal Research Committee. All experiments were carried out in accordance with the guidelines for the welfare of experimental animals issued by the U.S. Public Health Service Policy on Human Care and Use of Laboratory Animals and the University of California, Los Angeles (UCLA) Animal Research Committee. Wild-type C57BL/6J mice (20–30 g; Jackson Laboratory, Bar Harbor, ME, USA) of both sexes were used for these studies. Animals were 2–3 months old at the time of the experiments. Animals were deeply anesthetized with 1%–3% isoflurane (Abbott Laboratories, North Chicago, IL, USA) and euthanized by cervical dislocation. To prepare vertical cryostat sections of the retina, the eyecups were fixed in 4% paraformaldehyde (PFA) in 0.1 M phosphate buffer (PB), pH 7.4, for 15–60 min at room temperature (RT). Eyecups were then transferred to 30% sucrose in PB overnight at 4°C. The eyecups were embedded in optimal cutting temperature medium (Sakura Finetek, Torrance, CA, USA) and sectioned at 12–14 μm with a Leica CM3050S (Leica Microsystems, Buffalo Grove, IL, USA). Tissue sections were mounted onto gelatin-coated slides and sections were stored at −20°C until immunostaining.

### Immunostaining of Cryostat Sections of the Retina

Retinal sections were processed for immunohistochemical labeling using an indirect immunofluorescence method (Pérez de Sevilla Müller et al., 2013, 2015). Frozen retinal sections were thawed for 10–15 min at 37°C on a warming plate, then washed three times for 10 min each with 0.1 M PB (pH 7.4). Retinal sections were then incubated in 10% normal goat serum (NGS) and 0.3%–0.5% Triton X-100 in 0.1 M PB for 1–2 h at RT. Following removal of the blocking solution, sections were then placed in the primary antibodies (see Table 1), diluted in PB with 0.3%–0.5% Triton X-100 and 0.1% NaN_3_, overnight at 4°C. After incubation with the primary antibodies, the sections were washed three times for a total of 30 min in 0.1 M PB and placed in their corresponding secondary antibodies: Alexa Fluor goat anti-rabbit 488, goat anti-mouse 594 IgG, Alexa Fluor 568 goat anti-mouse IgG, or Alexa Fluor 568 goat anti-rat IgG (1:1000; Invitrogen, Grand Island, NY, USA) for 1–2 h at RT. The secondary antibodies were removed and sections were washed three times in 0.1 M PB for 10 min per wash. Sections were air-dried and mounted using Aqua Poly/Mount (Polysciences, Warrington, PA, USA), Vectashield (Vector Laboratories), or Citifluor (Citifluor, London, UK).

**Table 1:** **List of primary antibodies**.

Antibody	Host	Immunogen	Source	Dilution
Prox1	Rabbit	C-terminal 15 amino acids of mouse Prox1	BioLegend; San Diego, CA, USA PRB-238C	1:1000–1:2000
Glutamic Acid Decarboxylase 67 (GAD_67_)	Mouse	Amino acid residues 4–101 of human GAD67	EMD Millipore; Temecula, CA, USA MAB5406, AB_2278725	1:1000
Glycine	Rat	Glycine conjugated to paraformaldehyde and carrier protein thyroglobulin	ImmunoSolution; Everton Park, QLD, Australia; IG1002	1:1000
Calbindin	Mouse	Bovine kidney calbindin-D	Sigma-Aldrich; St. Louis, MO, USA C9848; clone CB-955	1:1000
Goα	Mouse	Bovine brain Go-alpha purified	Millipore; Temecula, CA, USA; MAB3073	1:300
Calretinin	Mouse	Recombinant human calretinin-22k	Swant; Bellinzona, Switzerland; Lot no 010399 clone 6B3	1:5000

All antibodies employed in this study have been used previously with PFA-fixed tissue; our immunostaining patterns in the mouse retina were identical to those previously reported in mouse or rat retina (Haverkamp and Wässle, 2000; Deng et al., 2001; Johnson et al., 2003; Martínez-Navarrete et al., 2008; Pérez de Sevilla Müller et al., 2013). Control experiments for nonspecific binding of the secondary antibodies were performed in both single and double-labeling studies.

### Whole-Mount Immunostaining

Whole-mounted retinas were processed for immunohistochemical labeling with a protocol similar to that used for the vertical sections. The retinas were removed from the eyecups and four small incisions were made on each retina to lay the tissue flat. Retinas were mounted onto nitrocellulose membrane filters, with the GCL facing upward (Millipore Corporation, Billerica, MA, USA), and fixed for 15 min in 4% PFA in 0.1 M PB at RT. The whole-mounted retinas were then washed in PB three times for a total of 90 min and incubated in 10% NGS with 0.3%–0.5% Triton X-100 at 4°C overnight. The retinas were subsequently incubated in primary antibody (see Table 1) for 7 days at 4°C and then washed three times for a total of 90 min in 0.1 M PB. The retinas were then placed in the appropriate secondary antibody overnight at 4°C. After three washes for a total of 90 min in PB, the retinas were mounted in Vectashield mounting medium (Vector Laboratories, Burlingame, CA, USA). Coverslips were sealed with nail polish and the slides were stored at 4°C protected from light.

### Antibodies

Retinal sections and whole mounts were processed with the following primary antibodies (Table 1) and dilutions: rabbit polyclonal antibody against Prox1 (1:1000–1:2000, PRB-238C, BioLegend, San Diego, CA, USA), mouse monoclonal antibody against calbindin (1:1000, C9848, cl. CB-955; Sigma-Aldrich, St. Louis, MO, USA), mouse polyclonal antibody against calretinin (1:5000, 010399 clone 6B3; Swant, Bellinzona, Switzerland), mouse monoclonal antibody to glutamic acid decarboxylase 67 (GAD_67_; 1:1000, MAB5406; Millipore, Temecula, CA, USA), mouse monoclonal antibody to Goα (1:300, MAB3073; Millipore, Temecula, CA, USA) and rat polyclonal antibody against glycine (1:1000; IG1002; ImmunoSolution, Everton Park, QLD, Australia). Prox1 antiserum was generated against the C-terminal 15 amino acids of mouse Prox1 (manufacturer’s technical information).

### Neurobiotin Injections into AII Amacrine Cells

Intracellular injections were performed as described previously (Pérez de Sevilla Müller et al., 2007, 2010a,b; Vuong et al., 2015). Borosilicate glass electrodes (#60200; A-M Systems; Sequim, WA, USA) were pulled and filled at their tips with 0.5% Lucifer Yellow (Sigma–Aldrich) 4% N-(2-aminoethyl)-biotinamide hydrochloride (Neurobiotin; Vector Laboratories, Burlingame, CA, USA), and back-filled with 0.1 M Tris buffer, pH 7.4. In retinal whole mounts, amacrine cell bodies located in the proximal INL at the border of the IPL were targeted for injection. Lucifer Yellow was iontophoresed (−1 nA) into a single cell body and when the bistratified morphology of the AII amacrine cell was recognized, the polarity of the current was reversed (+1 nA) and Neurobiotin was injected for 3 min. The retinas were then fixed in 4% PFA for 10 min and washed for 30 min in 0.1 M PB. Neurobiotin was visualized by incubating the retinas with the injected cells overnight at 4°C with streptavidin–FITC (1:500; Jackson ImmunoResearch, West Grove, PA, USA) in 0.1M PB containing 0.3% Triton X-100 (Sigma–Aldrich). Retinas were washed in PB three times for a total of 30 min. The retinas were subsequently processed for immunohistochemical staining.

### Fluorescent Image Acquisition

Immunostaining was evaluated with a Zeiss laser scanning microscope 710 or 880 (Zeiss LSM 710/Zeiss LSM 880; Carl Zeiss, Thornwood, NY, USA; RRID: SciEx_11637) with a Zeiss C-Apochromat 40× 1.2 NA corrected water objective or Zeiss C-Apochromat 63×/1.4 corrected oil objective at a resolution of 1024 × 1024 or 2048 × 2048 pixels. Images are presented as projection images of two to fifteen image scans (*z*-axis step between 0.3 and 1 μm). Confocal images were analyzed using Zeiss LSM 510 proprietary software (version 3.2). The intensity levels and contrast of the final images were adjusted in Adobe Photoshop CS2 v.9.02 (Adobe Systems, San Jose, CA, USA).

### Prox1 Amacrine Cell Density

Strongly expressing Prox1-immunoreactive amacrine cell density was determined from five whole-mounted retinas obtained from three mice (2 months old). Digital images for cell counting were collected at 100 μm intervals from the optic nerve head to the peripheral retina in the superior, inferior, temporal and nasal retinal quadrants. Three retinal fields (500 × 500 μm) per quadrant were collected for each retina using a Plan Apochromat 20×/0.8 NA corrected air objective with a 0.6 magnification factor. Cells were manually counted from the digital images using the cell counter in ImageJ^1^ to determine cell number and density. The total number of cells was determined in each sample area and expressed as the number of cells/mm^2^. Nearest-neighbor analysis (Wässle and Riemann, 1978) was performed on the cells located in the nasal area of the retina at 500 μm from the optic nerve head using the plugin “NND” in ImageJ.

For statistical testing we used a one-way ANOVA with *p* < 0.05 considered significant. Descriptive statistics and Gaussian fit were calculated using GraphPad Prism 4.0 (GraphPad Software, Inc, La Jolla, CA, USA) and presented as a mean ± standard deviation of the mean (SD).

## Results

### Prox1 Expression in the Adult Mouse Retina

**Figure 1:**
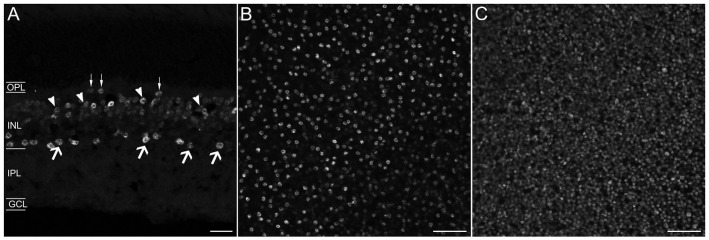
**Prox1 immunostaining in the mouse retina. (A)** Prox1 immunoreactivity in amacrine (arrows) and bipolar cell bodies (arrowheads) in the INL. Thin arrows show horizontal cells at the border of the OPL. **(B)** Prox1-immunoreactive amacrine cell bodies in the INL in a whole-mounted retina. **(C)** Prox1-immunoreactive bipolar cell bodies in the INL of a whole-mounted retina. *z*-step = 1 μm; 3–4 optical sections were compressed for viewing. OPL, Outer plexiform layer; INL, Inner nuclear layer; IPL, Inner plexiform layer; GCL, Ganglion cell layer. Scale bar **(A)**: 20 μm. **(B,C)**: 50 μm.

A polyclonal antibody that specifically recognizes the C-terminal 15 amino acids of mouse Prox1 (manufacturer’s technical information) labeled multiple cell bodies in the INL (Figure 1A). There were numerous Prox1-immunoreactive cell bodies in the proximal INL adjacent to the IPL that were similar in size and strongly immunostained (Figure 1A, arrows). Their location and size suggest that they are amacrine cells. There were also numerous weakly stained Prox1-immunoreactive cell bodies in the middle and distal INL; based on their size and position, they likely correspond to bipolar cells (Figure 1A, arrowheads). In addition, there were large and sparsely distributed Prox1-immunoreactive cell bodies in the distal INL at the border of the OPL (Figure 1A, thin arrows) that are horizontal cells (Belecky-Adams et al., 1997; Dyer et al., 2003; Cid et al., 2010). A few Prox1-immunoreactive somata were also in the GCL.

In whole-mounted retinas, strong Prox1 immunostaining was in cell somata in the proximal INL in all retinal regions (Figure 1B). These cell bodies were round in shape and their mean cell body diameter was 7.20 ± 0.44 μm (*n* = 100 cells; *N* = 5 retinas). Their cell diameter is consistent with their identity as amacrine cells in the rodent retina (Perry, 1981; Pérez de Sevilla Müller et al., 2007). Prox1 antibodies also labeled numerous somata in the middle and distal INL (Figure 1C). Overall, the immunolabeling of the cells in these regions of the INL was weaker compared to the immunolabeling of the amacrine cell bodies in the proximal INL. The weakly immunostained cell bodies were also round in shape, but their somal diameters were smaller, averaging 5.0 ± 0.3 μm (*n* = 100 cells; *N* = 5 retinas), consistent with their identity as bipolar cells (Ghosh et al., 2004; Pignatelli and Strettoi, 2004).

### Prox1-labeled Amacrine Cells are Glycinergic Cells

The majority of amacrine cells are either GABA or glycine immunoreactive, while a few amacrine cells do not contain either of these transmitters (for review see Vaney, 1990; Wässle and Boycott, 1991; Pourcho, 1996; Kay et al., 2011). To characterize the neurotransmitter used by Prox1-immunoreactive amacrine cells, we performed double-labeling experiments in retinal sections, using antibodies directed against GAD_67_, a GABA-synthesizing enzyme (Schnitzer and Rusoff, 1984), or glycine (Pourcho and Goebel, 1985).

**Figure 2:**
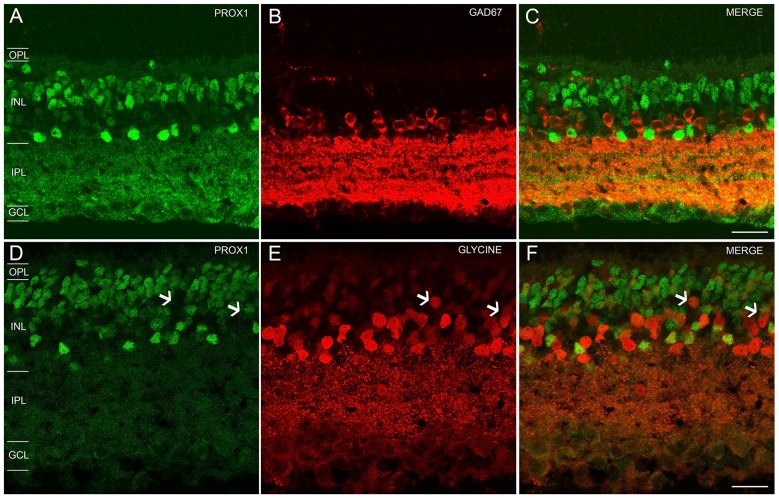
**Prox1, GAD_67_ and glycine immunoreactivity in the mouse retina. (A)** Prox1 immunoreactivity (green) in amacrine and bipolar cell bodies. **(B)** GAD_67_ (red) immunostained cell bodies in the INL and GCL. **(C)** Merged image shows that Prox1-expressing amacrine cells do not express GAD_67_ immunoreactivity. **(D)** Prox1 immunoreactivity (green) in amacrine and bipolar cell bodies. **(E)** Glycine-immunostained (red) cell bodies in the INL. **(F)** Merged image shows that many Prox1-expressing amacrine cells contain glycine immunoreactivity. Arrows indicate that not all glycine-immunoreactive bipolar cells contain Prox1 immunoreactivity. *z*-step = 0.5 μm; 2–3 optical sections were compressed for viewing. OPL, Outer plexiform layer; INL, Inner nuclear layer; IPL, Inner plexiform layer; GCL, Ganglion cell layer. Scale bar **(C,F)**: 20 μm.

In the GCL and proximal INL, numerous small-diameter somata were GAD_67_-immunoreactive (Figure 2B). However, the GAD_67_-immunoreactive amacrine cells did not contain Prox1 immunoreactivity (*n* = 0/87 cells; *N* = 4 retinas; Figures 2A–C).

In the INL, strong glycine immunostaining was found in multiple amacrine cells and weak immunostaining was observed in bipolar cells (Figures 2D–F), consistent with earlier findings (Menger et al., 1998; Vaney et al., 1998). Glycine immunoreactivity in bipolar cells is due to its diffusion from glycine-containing amacrine cells to bipolar cells through gap junctions (Vaney et al., 1998). All Prox1-immunoreactive amacrine cell bodies also contained glycine immunoreactivity (*n* = 131/131 cells; *N* = 5 retinas; Figure 2F); however, not all glycine-immunoreactive amacrine cells expressed Prox1 immunoreactivity (*n* = 31/87 cells; *N* = 2 retinas). Prox1-immunoreactive cell bodies comprise 35 ± 3% of all glycine-immunoreactive amacrine cells.

Together, these findings indicate that Prox1-expressing amacrine cells contain glycine immunoreactivity but not GAD_67_ immunoreactivity, consistent with Prox1-immunoreactive amacrine cells forming a subgroup of the glycinergic amacrine cells.

### Prox1 Amacrine Cells are AII Amacrine Cells

In the mouse retina, ~35% of the amacrine cells are glycine-immunoreactive (Voinescu et al., 2009; Zhang and McCall, 2012). The glycine-immunoreactive amacrine cells in the rat retina consist of at least eight narrow-field amacrine cell types. The most common type is the AII amacrine cell, which constitutes 20%–30% of the glycinergic amacrine cell population (Menger et al., 1998). On this basis, we tested if the Prox1 and glycine-immunoreactive amacrine cells were AII amacrine cells in the mouse retina.

To test for the expression of Prox1 immunoreactivity in AII amacrine cells, amacrine cells were randomly selected and injected with Lucifer Yellow and Neurobiotin (*N* = 3 retinas). Labeled AII amacrine cells (*n* = 5 cells) were identified by their distinct bistratified morphology and their thick lobules in the OFF sublayer of the IPL. The AII amacrine cells exhibited two different gap junctional couplings: AII amacrine to AII amacrine cells, and AII amacrine to bipolar cells (Famiglietti and Kolb, 1975; Strettoi et al., 1992; Chun et al., 1993; Urschel et al., 2006).

**Figure 3:**
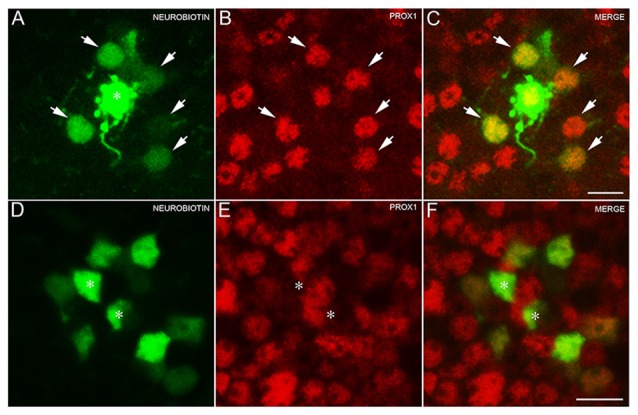
**AII amacrine cells labeling with Neurobiotin. (A)** An AII amacrine cell injected (asterisk) with Neurobiotin exhibits homologous coupling to other AII amacrine cells (arrows). **(B)** Prox1-immunoreactive amacrine cells in a retinal whole mount preparation (arrows). **(C)** Merged image shows that every dye-coupled AII amacrine cell body contains Prox1 immunoreactivity (arrows). *z*-step = 0.30 μm; 12 optical sections were compressed for viewing. **(D)** Bipolar cells coupled to the injected AII amacrine cell shown in **(A)**. **(E)** Weakly Prox1 immunostained bipolar cells in a retinal whole mount. **(F)** Merged image shows that Prox1 immunoreactivity is expressed by many but not all bipolar cell bodies (asterisks). *z-step* = 0.30 μm; 4 optical sections were compressed for viewing. Scale bar **(A,D)**: 10 μm.

The Neurobiotin-injected AII amacrine cell and every tracer-coupled AII amacrine cell were Prox1-immunoreactive (Figures 3A–C, arrows). Furthermore, no Prox1-immunostained cell bodies were in the immediate vicinity of the Neurobiotin-injected AII amacrine cell, suggesting that AII amacrine cells are the predominant cell type expressing Prox1.

In addition, only some of the AII amacrine cell coupled bipolar cells were not Prox1-immunoreactive, indicating that not all bipolar cell types express Prox1 (Figures 3D–F, asterisks). These results are consistent with immunohistochemical findings of subpopulations of glycine-immunoreactive bipolar cell bodies lacking Prox1 immunoreactivity (Figures 2D–F, arrows).

**Figure 4:**
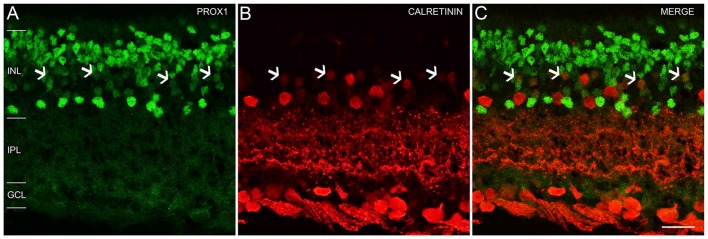
**Prox1 and calretinin immunoreactivity in the mouse retina. (A)** Prox1 immunoreactivity (green) in amacrine and bipolar cell bodies. **(B)** Calretinin-immunostained (red) cell bodies in the INL and GCL. **(C)** Merged image shows that Prox1 expressing amacrine cells are separate from calretinin-immunoreactive amacrine cells. Arrows indicate examples of some cell bodies in the middle INL that were weakly immunostained for Prox1 and calretinin. *z*-step = 1 μm; 2 optical sections were compressed for viewing. INL, Inner nuclear layer; IPL, Inner plexiform layer; GCL, Ganglion cell layer. Scale bar **(C)**: 20 μm.

An earlier study of the adult mouse retina reported Prox1 expression in weakly immunostained calretinin amacrine cells (Cid et al., 2010). We tested if Prox1 immunoreactivity is in calretinin-expressing amacrine cells. The Prox1-immunoreactive cell bodies in the proximal INL adjacent to the IPL did not contain calretinin immunoreactivity in retinal sections (Figures 4A–C). These findings are consistent with other studies that showed that AII amacrine cells in the mouse retina are not calretinin-immunoreactive (Haverkamp and Wässle, 2000; Vuong et al., 2015; Meyer et al., 2016). Some cell bodies in the middle INL were weakly immunostained for Prox1 and calretinin (Figures 4A–C, arrows), which is consistent with an earlier study (Cid et al., 2010).

### Prox1 Expression in Other Retinal Cell Types

**Figure 5:**
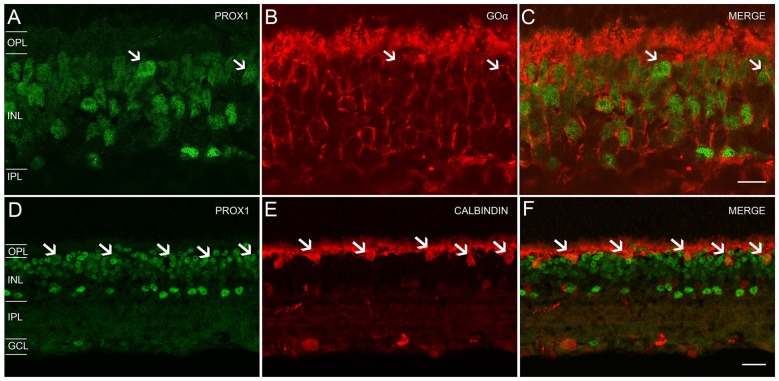
**Prox1, Goα and calbindin immunoreactivity in the mouse retina. (A)** Prox1-immunoreactive (green) amacrine and bipolar cell bodies. **(B)** Goα immunoreactivity (red) in ON-type bipolar cells. **(C)** Merged image shows that Goα-immunoreactive bipolar cells express Prox1 immunoreactivity, but not all Prox1-immunoreactive cells are Goα-immunoreactive (arrows). *z*-step = 0.5 μm; 3 optical sections were compressed for viewing. Scale bar **(C)**: 10 μm. **(D)** Prox1-immunoreactive (green) amacrine, bipolar and horizontal cell bodies (arrows). **(E)** Calbindin immunoreactivity (red) in horizontal cell bodies (arrows). **(F)** Merged image shows that horizontal cells contain Prox1 immunoreactivity (arrows). *z-step* = 1 μm; 2 optical sections were compressed for viewing. OPL, Outer plexiform layer; INL, Inner nuclear layer; IPL, Inner plexiform layer; GCL, Ganglion cell layer. Scale bar **(F)**: 20 μm.

Our experiments with glycine antibodies indicate that some, but not all, bipolar cells displayed weak Prox1 immunoreactivity (Figures 2D–F). Studies in the postnatal and adult mouse retina (Dyer et al., 2003; Cid et al., 2010) showed weak Prox1 immunostaining in bipolar cells. Building on these findings, we performed double labeling experiments for Prox1 and Goα, a marker for ON-cone bipolar cells and rod bipolar cells in the mouse retina (Vardi, 1998; Haverkamp and Wässle, 2000). All Goα-expressing bipolar cells exhibited weak Prox1 immunoreactivity. Prox1-immunolabeled cell bodies that lacked Goα-immunoreactivity were also observed (Figures 5A–C, arrows). Together, these experiments indicate that ON-cone and rod bipolar cells are the predominant bipolar cell types that express Prox1. In addition, some Prox1-expressing bipolar cells are likely OFF-cone bipolar cells, based on the lack of Goα immunostaining (Vardi, 1998; Haverkamp and Wässle, 2000).

We confirmed Prox1 expression in horizontal cells using antibodies to Prox1 and calbindin, a specific marker for horizontal cells (Röhrenbeck et al., 1987; Chun and Wässle, 1993; Massey and Mills, 1996; Haverkamp and Wässle, 2000; Hirano et al., 2005, 2011). Large Prox1 and calbindin-immunostained somata were located in the distal INL at the OPL border, consistent with their identity as horizontal cells (Figures 5D–F, arrows).

### Prox1 Amacrine Cell Distribution

**Figure 6:**
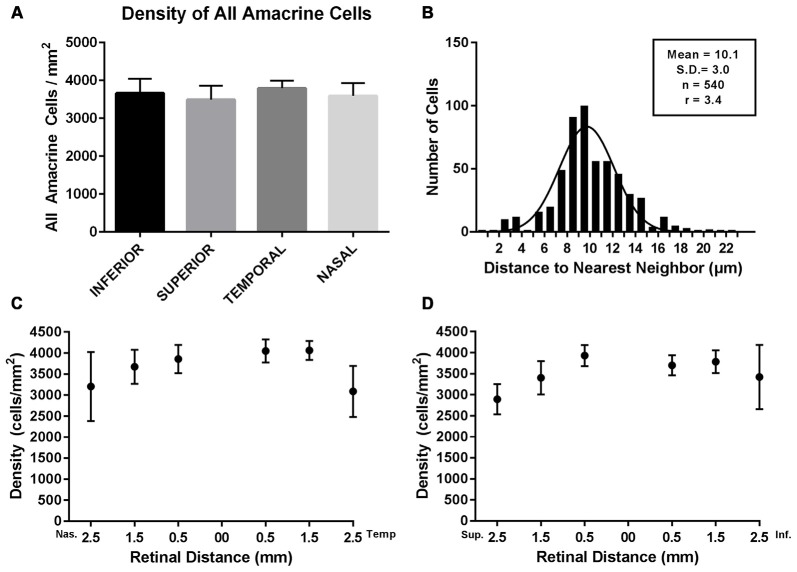
**Density and distribution of Prox1-immunoreactive AII amacrine cells. (A)** Prox1-immunoreactive AII amacrine cells were distributed across all four retinal quadrants of the adult mouse retina. **(B)** Histogram of the nearest-neighbor distances of the Prox1-immunoreactive cell bodies. Solid line shows the Gaussian fit to the distribution of nearest-neighbor distances. The mean nearest-neighbor distance, standard deviation, total number of cells and the regularity index (RI) are indicated. **(C)** Density of Prox1-immunoreactive cell bodies in nasal and temporal retina. **(D)** Density of Prox1-immunoreactive cell bodies in superior to inferior retina. **(C,D)** Density of Prox1-immunoreactive cell bodies was counted in whole-mounted retinas from the optic nerve head to peripheral retina.

The density of Prox1-immunoreactive amacrine cells was measured from the superior to inferior retina and from the nasal to temporal retina in whole-mount preparations (*N* = 5). Prox1-immunoreactive amacrine cell bodies were in all regions of the retina, with little variation in their distribution and no significant differences (*P* = 0.556, one-way ANOVA; *N* = 5 retinas) between the different retinal quadrants (Figures 6A,C,D). The highest average Prox1-containing amacrine cell density (3887 ± 160 cells/mm^2^) was found 0.5 mm from the optic disc. Cell density (3717 ± 152 cells/mm^2^) was only slightly lower at 1 mm from the optic nerve head. The mid-peripheral retina, 2 mm from the optic nerve head, had a lower cell density (3133 ± 350 cells/mm^2^).

In addition, we evaluated the regularity of the mosaic of Prox1-immunoreactive amacrine cells (Figure 6B). We measured the nearest-neighbor distance for each cell body in an area of 250 × 500 μm in the nasal retinal quadrant, 0.5 mm from the optic disc. The area contained a total of 540 Prox1-immunoreactive amacrine cells and their mean nearest-neighbor distance was 10.1 ± 3 μm (mean ± SD). The frequency of nearest-neighbor distances fits a Gaussian distribution, indicating that the Prox1-immunoreactive cell bodies formed a regular mosaic in this region. Furthermore, their regularity index (RI), measured as the ratio between the mean of the nearest-neighbor distances and its standard deviation, was 3.4 (Eberhardt, 1967; Wässle and Riemann, 1978). Together these findings suggest that Prox1-labeled amacrine cells comprise a single amacrine cell population.

## Discussion

In the mouse retina, the transcription factor Prox1 is strongly expressed in a single row of amacrine cell bodies in the INL at the IPL border, corresponding to AII amacrine cells. Horizontal cell bodies were also immunolabeled, together with numerous cell bodies in the middle and distal INL, likely bipolar cells. These findings are overall consistent with previous studies of the neuronal expression of Prox1 immunoreactivity in the vertebrate retina (Belecky-Adams et al., 1997; Dyer et al., 2003; Edqvist and Hallböök, 2004; Fischer et al., 2007).

Our immunohistochemical experiments demonstrated that Prox1-immunoreactive amacrine cells label ~35% of the narrow-field glycine-immunoreactive cells. In addition, intracellular labeling with Neurobiotin directly demonstrated that Prox1 immunoreactivity is localized to AII amacrine cells. Together, these results strongly support the idea that Prox1 immunoreactivity is selectively found in AII amacrine cells in the mouse retina. These findings are also consistent with studies in the rat retina that demonstrated that Prox1 immunoreactivity is expressed in parvalbumin-containing AII amacrine cells (Dyer et al., 2003).

Our findings are in contrast to a report that Prox1 immunoreactivity is in the majority of calbindin-immunoreactive cells in the adult mouse retina (Cid et al., 2010). Our experiments with calbindin antibodies never showed colocalization with Prox1 immunoreactivity in the proximal INL (Figures 5D,E). These differences in immunostaining may be due to differences in the specificity of the Prox1 antibodies used in each study. The Prox1 antibody used in our study was directed to the C-terminal 15 amino acids of mouse Prox1 (see “Materials and Methods” Section). Furthermore, the Prox1 antibody used in the earlier study of the rat retina that showed Prox1 expression in parvalbumin-containing AII amacrine cells was also directed to the C-terminus of Prox1 (Dyer et al., 2003). In contrast, the specificity of the Prox1 antibody used in the colocalization study with calbindin in the mouse retina is not given (Cid et al., 2010). Other, although less likely reasons for these differences in the localization of Prox1 immunoreactivity are the age and genetic backgrounds of the mice used in these studies.

### Distribution and Density of AII Amacrine Cells

Prox1-immunoreactive cell body density did not vary between the different retinal quadrants (*P* = 0.556, one-way ANOVA; *N* = 5 retinas). Prox1-immunoreactive cell density was highest (3887 ± 160 cells/mm^2^) 0.5 mm from the optic nerve head and there was a slight reduction of cell density (3717 ± 152 cells/mm^2^) 1 mm from the optic nerve head. Cell density (3133 ± 350 cells/mm^2^) was approximately 20% lower in mid-peripheral retina, 2 mm from the optic nerve head. Our estimates of AII amacrine cell density differ from an earlier report based on Disabled 1 immunostaining of AII amacrine cells (Rice and Curran, 2000); Disabled 1-immunoreactive cell density (4086 cells/mm^2^) was highest in central retina and lowest (1560 cells/mm^2^) in dorsal peripheral retina. Although peak cell densities are similar overall, the lower cell density of Disabled 1-immunoreactive cells in the dorsal peripheral retina may be due to an absence or very low levels of Disabled 1 expression. The lower cell density of Disabled 1-immunoreactive cells could also be due to tissue preparation or immunostaining protocols.

The AII amacrine cell distribution based on Prox1 immunostaining in the mouse retina is similar to the AII amacrine cell distribution in other mammalian retinas. In mouse, as well as in rat, rabbit, cat, macaque and human retinas, AII amacrine cell density was greatest in central retinal regions and lowest in the peripheral retina (Vaney, 1985; Wässle et al., 1995; Massey and Mills, 1999; Lee et al., 2004). Our studies of the mouse retina showed a ~20% decrease in cell density between central to mid-peripheral retinal regions. In contrast, there is a ~60%–80% decrease in cell density in rat, rabbit, cat, macaque and human retinas, depending on the species (Vaney, 1985; Mills and Massey, 1999; Lee et al., 2004). The difference of mouse AII amacrine cell density in central and mid-peripheral retinal regions is similar to the modest difference between central and peripheral retinal densities of VIP-Cre-expressing and ChAT-immunoreactive amacrine cells (Keeley et al., 2007; Pérez de Sevilla Müller et al., in press). TH-immunoreactive amacrine cell density does not vary between the central and peripheral mouse retina (Versaux-Botteri et al., 1984; Whitney et al., 2009; Keeley et al., 2014).

Assuming ~39,700 amacrine cells/mm^2^ in the INL of the C57BL/6 retina (Jeon et al., 1998), we estimate that AII amacrine cells comprise ~9.8% of the total amacrine cell population. This percentage is similar to the proportion of AII amacrine cells found in other mammalian retinas. For instance, using parvalbumin as a marker, AII amacrine cells are estimated to make up 10% of the amacrine cells in the rat retina (Wässle et al., 1993) and 11% of amacrine cells in the rabbit retina (Casini et al., 1995; Strettoi and Masland, 1996; Massey and Mills, 1999). Using calretinin as a marker, AII amacrine cells account for nearly a quarter of all amacrine cells in the cat retina (Vaney, 1985; Macneil et al., 2009) and 11% of all amacrine cells in the macaque retina (Wässle et al., 1995; Mills and Massey, 1999).

The AII amacrine cell population forms the largest identified amacrine cell population in the mouse retina, accounting for ~10% of the total amacrine cell population. In comparison, the ChAT- and VIP-immunoreactive amacrine cell populations in the INL account for ~3.0%–5.0% and ~1.4% of the amacrine cell population, respectively (Jeon et al., 1998; Whitney et al., 2008; Keeley et al., 2014; Pérez de Sevilla Müller et al., in press). Furthermore, the dopamine-containing or TH-immunoreactive amacrine cell population is considerably smaller with ~450–600 cells per retina in the C57BL/6J mouse strain (Versaux-Botteri et al., 1984; Masland et al., 1993; Gustincich et al., 1997; Whitney et al., 2009; Keeley et al., 2014).

Prox1-immunoreactive amacrine cell bodies form a nonrandom mosaic, suggested by the fit of their nearest-neighbor distance distribution to a Gaussian distribution (Wässle and Riemann, 1978). Their RI of 3.4 is indicative of a regular distribution of cell bodies in the Prox1-immunoreactive amacrine cell mosaic; a ratio of 1.0 indicates a random distribution and higher ratios indicate a more regular distribution (Eberhardt, 1967; Wässle and Riemann, 1978). The RI of Prox1-immunoreactive amacrine cells in the mouse retina is similar to the regularity indices of AII amacrine cells in rat (RI = 5.1, Wässle et al., 1993; Lee et al., 2004), rabbit (RI = 3.23, Casini et al., 1995), cat (RI = 3.55, Vaney, 1985; Lee et al., 2004), monkey (RI = 2.7, Wässle et al., 1995) and human (RI = 3.76, Lee et al., 2004) retinas.

In conclusion, we report that Prox 1 immunoreactivity can be used as a marker to identify AII amacrine cells in the mouse retina. On this basis, Prox1 antibodies can be used to identify AII amacrine cells to study AII amacrine cell number and distribution in experimental and genetic (Keeley et al., 2014; Reese and Keeley, 2016) models. Additionally, the Prox1 promoter can be useful for developing genetic tools to label a subpopulation of retinal neurons, including AII amacrine cells to study AII amacrine cell structure, connectivity and physiology.

## Author Contributions

NCB and LPSM conceived the project, and designed the experiments; LPSM, SSA and JS performed the experiments and analyzed the data; LPSM, SSA, JS, NCB wrote the article; LPSM and NCB supervised the project.

## Funding

Support for these studies are from NIH R01 EY04067 (NCB) and NIDDDK P30 DK41301 (UCLA Cure Center Core). This work was supported in part by Career Scientist Award (14F-RCS-004) from the United States Department of Veterans Affairs. The contents do not represent the views of the U.S. Department of Veterans Affairs or the United States Government. NCB is a VA Career Research Scientist.

## Conflict of Interest Statement

The authors declare that the research was conducted in the absence of any commercial or financial relationships that could be construed as a potential conflict of interest.

## Footnotes

^1^http://rsb.info.nih.gov/ij/index.html
